# Factors Influencing Germination of a Functionally Important Grassland Plant, *Iris tenax*

**DOI:** 10.1371/journal.pone.0090084

**Published:** 2014-02-28

**Authors:** Katherine D. Jones, Thomas N. Kaye

**Affiliations:** 1 Department of Botany and Plant Pathology, Oregon State University, Corvallis, Oregon, United States of America; 2 Institute for Applied Ecology, Corvallis, Oregon, United States of America; University of Marburg, Germany

## Abstract

Grassland prairies of western Oregon and Washington are among the most endangered ecosystems in the United States. Active management and restoration are needed to promote biodiversity in the region. To support plant production for use in habitat restoration, we developed germination protocols for greenhouse propagation of *Iris tenax* (Oregon iris). Dormancy was most effectively overcome (63% germination) by four weeks of warm stratification at 20/30°C followed by 6–12 weeks of cold stratification at 5°C suggesting that *I. tenax* may have morphophysiological dormancy. This result was consistent across multiple source populations.

## Introduction

Native prairies of the Pacific Northwest have experienced significant reduction in size since European settlement of the region; the Willamette Valley in western Oregon has lost an estimated 99.5% of its pre-settlement prairie habitat [1] while the Puget Trough grasslands have been reduced to 5–10% of the their historic extent [2] making these prairies some of the most highly endangered ecosystems in the United states [3]. Prior to European settlement, this region was a mosaic of coniferous forests, oak savannas and grassland prairies with high plant and animal diversity. Habitat loss, caused by conversion to agriculture, urbanization and natural succession to shrubland and forest due to loss of disturbance regime has reduced native habitats to a fraction of their pre-settlement extent [4]. Due to severe fragmentation, remaining native grasslands and the populations of species within them are frequently too small with too little genetic diversity to be sustainable [5]–[7]. Active management, including vegetation manipulation, native species reintroduction and population augmentation is necessary to restore these habitats and species [8]–[11].

*Iris tenax* Douglas ex Lindl (Oregon iris, plant nomenclature follows [12]) is a rhizomatous perennial found throughout this region and is a likely candidate for inclusion in habitat restoration because of its potential value as a nectar resource for endangered butterflies from western Washington to northern California. In past restoration efforts, this species has exhibited poor establishment from seed, so its restoration at new sites is most effective from container-grown stock [13]. Members of the genus *Iris* frequently have some type of dormancy [14]–[16], and lack of an established protocol for germinating seeds of *I. tenax* impedes efficient container production of this species for restoration plantings.

The genus *Iris* is described as having demonstrated dormancy in many species [17], including mechanical dormancy [15], [18], physiological dormancy [14], [19], morphological, and morphophysiological dormancy [20]–[22]. Germination studies conducted on wetland and ornamental *Iris* species have identified cold stratification [23], [24], warm stratification [19], chemical scarification [25] and physical scarification [15] as methods of breaking dormancy. Few studies though have investigated dormancy among upland *Iris* species (see [26] for an exception with a desert species, *I. atrofusca*). Germination of *I. tenax*, in particular, has not been previously described and, because of the range of dormancy types found in the genus *Iris*, it is difficult to predict germination requirements for this species from grasslands on well-drained soils.

We conducted a series of laboratory experiments to develop dormancy breaking techniques for *I. tenax*. We focused on stratification temperatures and durations in our experimental treatments because physiological dormancy is common in this geographic region and stratification often breaks this form of dormancy.

## Materials and Methods

We conducted three separate experiments involving *I. tenax* seeds. The first examined warm and cold stratification treatments alone and in combination, and the second applied short periods of dry heat followed by cold stratification. The third tested seeds from several wild populations to evaluate the generality of conclusions from the first test. We focused on warm and cold stratification treatments because these are relatively common methods of breaking dormancyin native plants in the geographic region of this species [27]. Because this work was exploratory, we applied a wide range of both warm and cold stratification periods, with the longest periods matching what could be expected under wild field conditions. The initial stratification experiments took place in 2010, followed in 2011 by the after ripening and source population experiments.

Temperature controlled rooms at the Oregon State University Seed Laboratory were used to stratify and germinate the seeds. Following treatments in all three experiments, seeds were placed in a germination chamber with alternating 15/25°C temperatures and 8/16 hour photoperiods (8 hours of warm light, 16 hours of cold dark). We determined a seed to have germinated if, after two weeks, the radicle emerged at least 2 mm beyond the seed coat. Development of mold on seeds was common during both the stratification and germination phases; in many cases seeds with mold still germinated, so moldy seeds were not removed from germination boxes over the course of the treatments. Percentage of live seeds (Table 1) was determined by staining two replicates of 60 seeds from each source with Tetrazolium chloride (TZ). Seeds were cut in half to expose the embryo then soaked in TZ solution for 36 hours following standard methods [28].

**Table 1:** Seed sources of *I. tenax*.

Source Name	Type	Elevation (meters)	Estimated live seed % (±SE)	Storage duration
Coast Range (CR)	Native Population	1067	60 (5)	3 months
Heritage Seedlings (HS)	Commercial/mixed accession; Marion, Polk and Benton Co.	146	65.8 (2.7)	1 year
Mehema (M)	Native Population	155	62.2 (1.8)	2 months
Pigeon Butte (PB)	Native Population	144	40 (2)	3 months
South Santiam (SS)	Native Population	260	45.5 (5.5)	3 months
Silver Falls Seed (SF)	Commercial/mixed accession; Marion Co.	135	41.2 (3.8)	2 years

Estimated live seed based on visual estimate of embryo staining using tetrazolium chloride. Embryos of seeds from the Pigeon Butte population did not stain uniformly which may have led to an underestimate of viability. All seeds harvested prior to 2011 were stored consistent with commercial seed standards.

### Stratification

Seeds for this experiment were provided by Heritage Seedlings Inc. (Salem, Oregon, Table 1) and were from mixed-accession production beds developed from wild Willamette Valley populations. We tested combinations of warm and cold wet stratification in a 3×7 factorial design, for a total of 21 treatment combinations. Each treatment combination was replicated four times with 50 seeds in each replicate. Each replicate was placed on germination paper moistened with distilled water in a 12 cm×12 cm plastic box with a tight fitting lid. Treatment groups for warm stratification (20/30°C, 8 hrs of fluorescent grow-light, 16 hrs of dark) were control (seeds received no warm stratification), 2 week and 4 week stratification groups. Following warm stratification, seeds either received no further manipulation (controls) or were given cold stratification (constant 5°C); treatment groups for cold stratification were control, 2, 4, 6, 8, 10, and12 week treatment periods. Following stratification, seeds were placed in a germination chamber and total germination was recorded after two weeks. We added distilled water throughout the experiment as needed to maintain moisture in each replicate.

### After ripening

We tested the effects of short intervals of heat applied to dry seeds, followed by cold stratification to determine if a relatively lengthy warm stratification process (up to 4 weeks) could be replaced by a short dry-heat treatment, potentially speeding up the cultivation process. Seeds for this experiment were from Heritage Seedlings Inc., the same source and seed lot as those used in the wet stratification experiment.

We tested a total of nine treatment combinations in a 3×3 factorial design. Heat was applied in three treatments, 40°C for 24, 40°C for 48 hours and 50°C for 24 hours. All heat treatments were followed by cold (5°C) stratification for one of three treatment periods, control (0), 4 or 8 weeks. Each treatment combination was replicated 4 times using 50 seeds for each replicate and germination was recorded after two weeks.

### Stratification across multiple source populations

**Figure 1:**
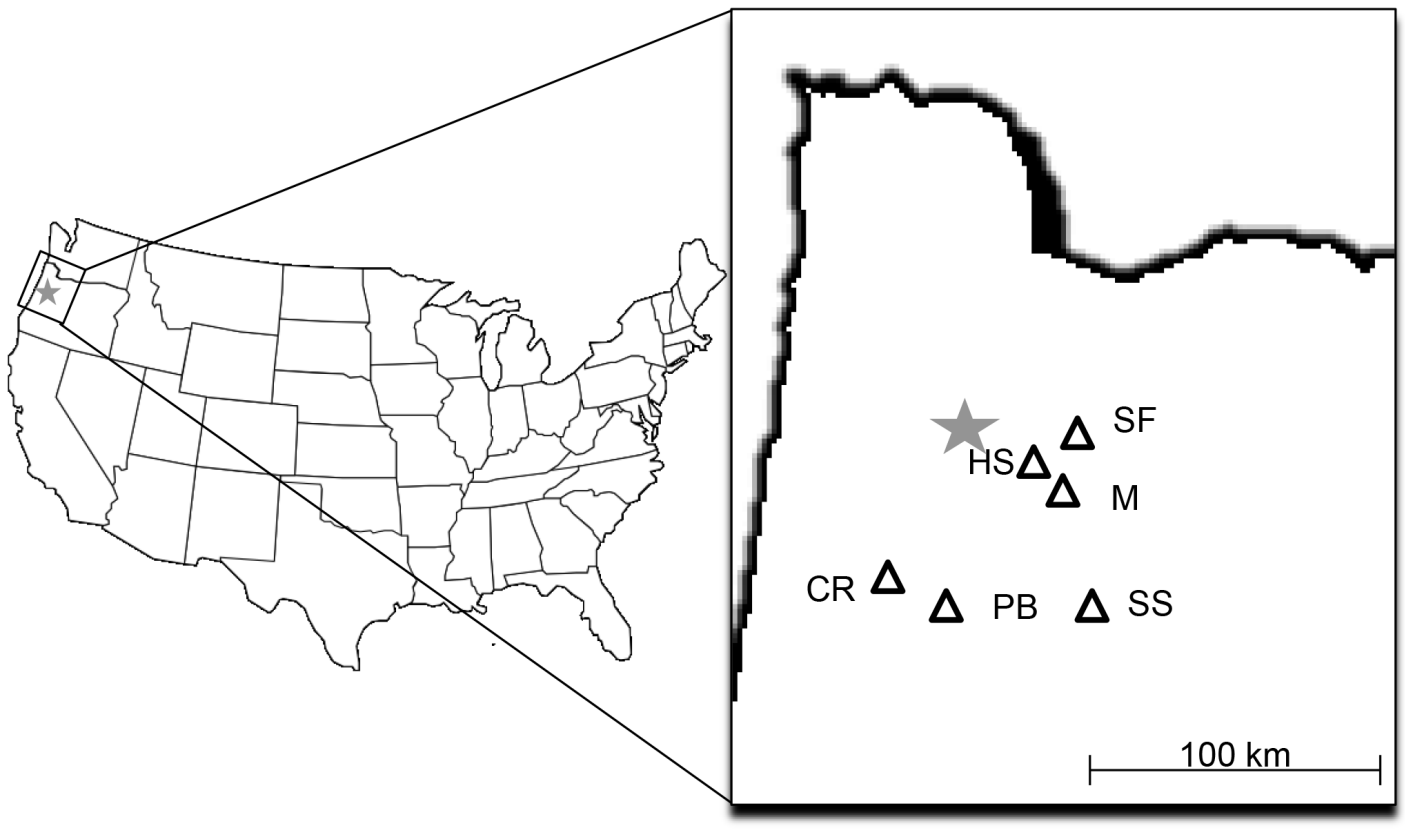
*I. tenax* source locations in and around western Oregon, USA.

To determine if dormancy breaking mechanisms are consistent across the range of this species, we used seeds from six sources in western Oregon. Seeds were collected from four wild populations and from two commercial sources. With the exception of Pigeon Butte, all seeds used in this experiment were purchased from or donated by professional growers/collectors. Wild collection occurred on private and/or public land and was covered by permits held by those collectors. The seeds from the Pigeon Butte population were collected by the authors from Finley National Wildlife Refuge in accordance with a special use permit from USFWS held by the Institute for Applied Ecology. These seed sources represented a range of populations that are likely to be used in restoration projects in this region (Table 1 and Figure 1). By including wild and commercially available seeds we tested the range of seeds that would be available to land managers working on habitat restoration projects in the region.

We tested the effects of warm and cold stratification in a 2×2 factorial design replicated across six seed sources. Warm stratification (20/30°C) was applied for either 1 or 4 weeks followed by cold stratification (5°C) for 4 or 8 weeks. Germination procedures followed those described for stratification experiments, above. Because seed availability was low we tested a limited number of treatment combinations based on results of the stratification and after ripening experiments.

### Statistical analysis

We used two-way analysis of variance (ANOVA) to test for the effects of warm and cold stratification periods on mean germination of *Iris tenax* in each experiment separately. In each analysis, the dependent variable was the proportion of seeds in each replicate that had germinated at the end of the two week period in the germination chamber. Warm and cold stratification were independent variables that were tested for their main and interaction effects on germination. In all cases, the proportion of germinated seeds was calculated as the number of seeds with radicle or leaf emergence divided by the total number of seeds tested (50). We did not adjust the number of seeds tested nor the calculation of percent germinated to account for estimated viability of seed lots. We also conducted pairwise comparisons between each treatment group using a Tukey honestly significant difference (HSD) test. No transformations were necessary to meet the assumptions of either the ANOVA or the Tukey HSD test.

## Results

### Stratification

**Figure 2:**
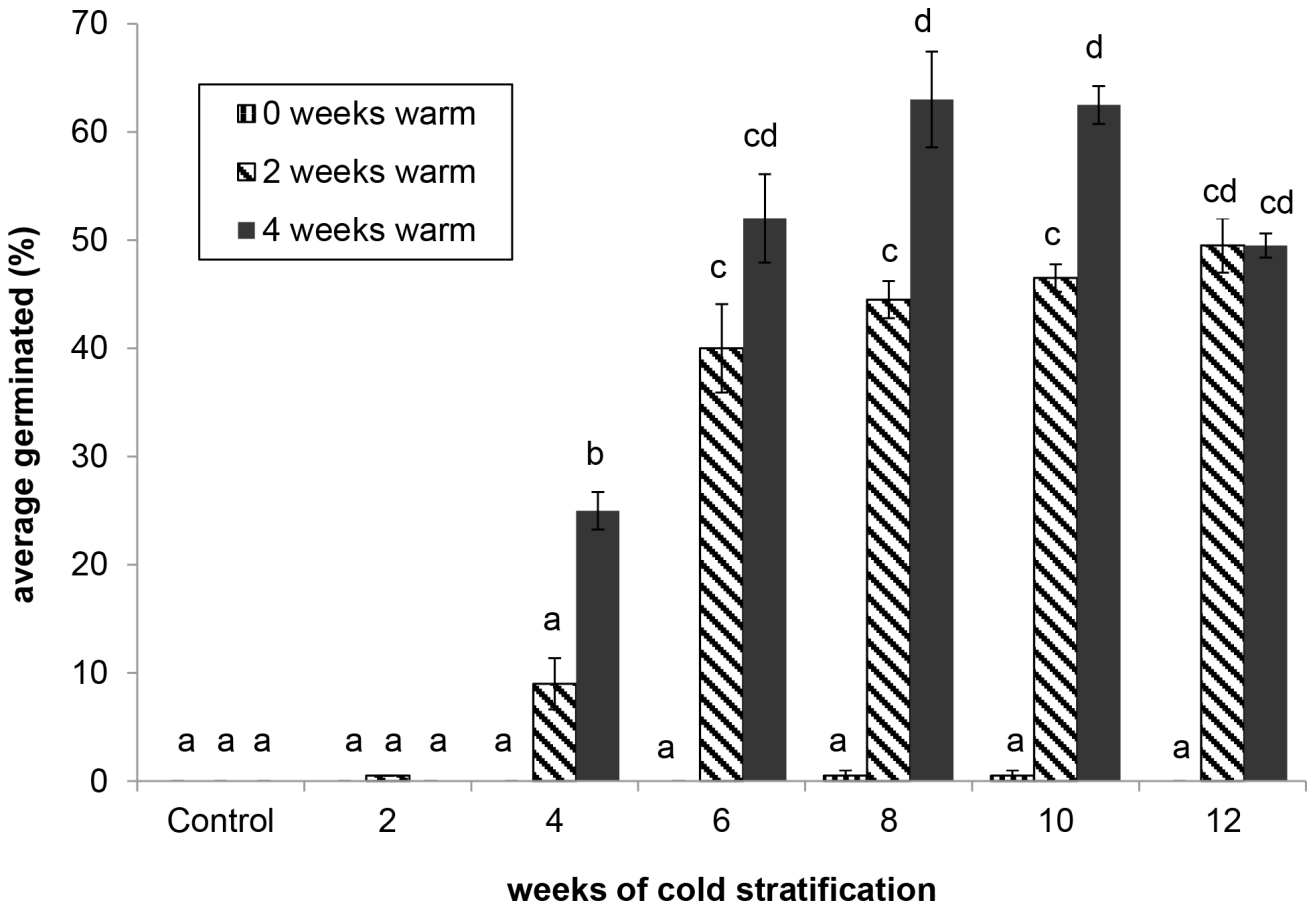
Mean germination of *Iris tenax* seeds after 2 and 4 weeks warm, wet stratification followed by cold, wet stratification. Error bars represent one standard error of the mean, bars with the same letter represent means that did not differ significantly (p≤0.05) based on Tukey HSD.

Both warm and cold stratification significantly affected germination (*F* (1,80) = 124.2, *p*<0.001 and *F* (1,80) = 134.9, *p*<0.001 respectively from ANOVA F-test) and there was a statistically significant interaction between the two treatments (*F*(1,80) =  51.4, *p*<0.001 from ANOVA F-test). Mean germination was greatest after 4 weeks of warm stratification followed by cold stratification for 8 or 10 weeks (Figure 2). Though the greatest mean germination occurred after 8 and 10 weeks of cold stratification (63%±4.4% SE and 62.5%±1.8% SE respectively) there was no statistically significant difference between these treatment groups and the 6 and 12 week cold stratification treatments (52.0%±4.1% SE and 49.5%±1.1% SE).

### After ripening

Dry heat did not result in any germination in the treatment combinations we tested.

### Stratification across multiple source populations

**Figure 3:**
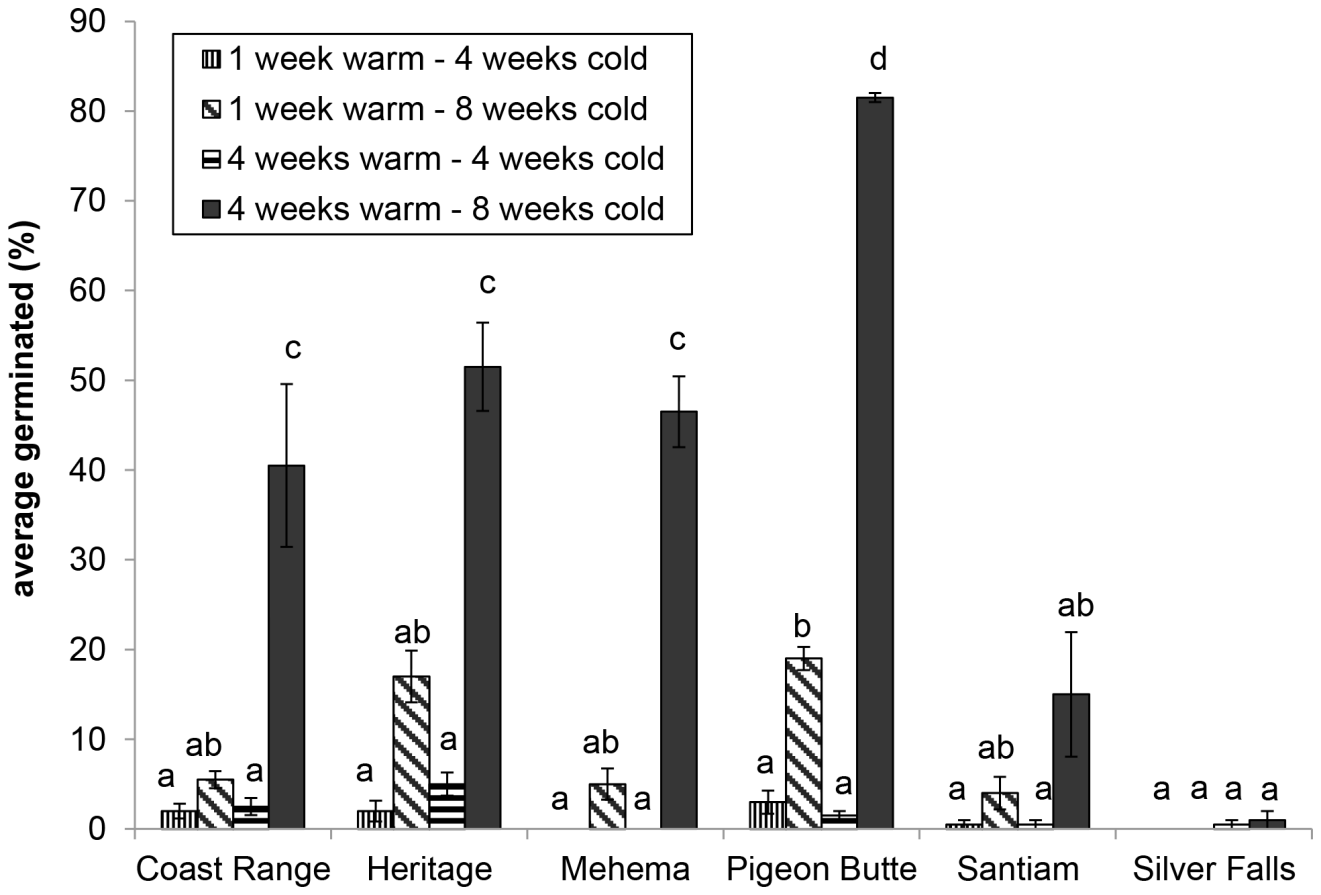
Mean germination versus treatment combination for six different source populations of *Iris tenax*. Error bars represent one standard error of the mean, bars with the same letter represent means that did not differ significantly (p≤0.05) based on Tukey HSD.

With only one exception, all of the source populations we tested had the greatest germination after 4 weeks of warm stratification followed by 8 weeks of cold stratification (Figure 3). Mean germination with this combination of treatments ranged from 15% (Santiam) to 80% (Pigeon Butte) with seeds from all sites except Silver Falls, which had low germination (<2%) in all treatment combinations.

## Discussion

Highest germination of *I. tenax* was achieved by applying 4 weeks of warm stratification followed by 6–12 weeks of cold wet stratification. Though we did not specifically test for dormancy types, the treatments that broke dormancy in *Iris tenax* seeds are consistent with descriptions of deep simple morphophysiological dormancy [16], [29]. Morphological and physiological dormancy of *I. virginiana* is also overcome by a combination of both warm and cold stratification. Morgan [19] germinated seeds of *I. virginiana* by applying cold stratification followed by warm stratification. Few studies have reported this type of dormancy in *Iris* but it is possible that morphophysiological dormancy is present in other species in this genus.

**Figure 4:**
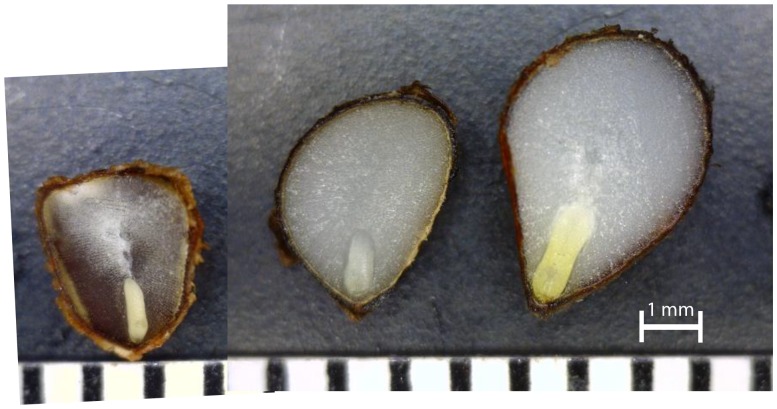
Embryos of *Iris tenax* dry seed (left), after 3 weeks warm stratification (middle) and just before emergence ∼12 weeks total stratification (right). One hatch mark  = 1 mm.

Some species in the family Iridaceae have seeds with underdeveloped or linear embryos requiring a period of maturation and growth inside the seed prior to germination, a characteristic of morphological dormancy (reviewed in [17]). The embryo of a mature seed of *I. tenax* is only about 1 mm long, ¼ the length of the whole seed. The embryo nearly quadruples in size before germination occurs (Figure 4) suggesting that further development and likely cellular differentiation of the embryo within the seed is necessary prior to radicle emergence, [16]. Cold stratification is necessary for germination suggesting that inhibitors in the seed, likely in the endosperm [14], [30], [31] or embryo [32] may be present in this species and may need to be leached out before radicle emergence occurs, a trait of physiological dormancy. Although the seed coat can mechanically restrict germination in some *Iris* species [15], [31], seed coat removal is ineffective at releasing *I. tenax* seeds from dormancy [13]. Giberellic acid is also ineffective at overcoming dormancy in this species, suggesting deep dormancy in its seeds [13]. Morphophysiological dormancy is often overcome through a combination of warm and cold stratification treatments [16]. Based on these processes observed in other members of the Iridaceae and on our observations of embryo development in *I. tenax*, we suggest that during warm stratification the embryo of *I. tenax* elongates and cells differentiate in preparation for emergence and during cold stratification, germination inhibitors are leached from the seed.

Dormancy in seeds from temperate latitudes may be an adaptive function to prevent seeds from germinating during unfavorable seasons [33]. The warm and cold temperature sequences we observed to be effective at releasing seeds from dormancy in *I. tenax* in the laboratory may be similar to the natural environmental conditions that regulate its germination in the wild. This species blooms in late spring with fruit maturation typically occurring in June. Following dispersal in July, seeds are subjected to warm summer temperatures. Though summer is a dry period in western Oregon [34], residual soil moisture coupled with high temperatures may be sufficient to provide warm stratification, stimulating development of the embryo within the seed, especially in years with occasional late summer rains. The slow leaching of inhibitors throughout the cool wet winter, followed by warming temperatures in early spring, may allow emergence of the radicle and shoot under favorable spring conditions the year following seed dispersal.

Seed dormancy may be under genetic control [35], [36]. In some species, dormancy differs among genotypes and with varied environmental conditions [37]–[39]. This variety within a species has been demonstrated in *I. hexagona* where dormancy differs depending on the level of salinity to which the maternal plant is exposed [40]. Dormancy in *I. tenax* however, appears to be relatively consistent throughout the species' geographic range in Oregon. With only one exception, germination was highest for seeds that received 4 weeks of warm stratification followed by 8–10 weeks of cold stratification for each seed source tested. The seed from Silver Falls, with mean germination less than 2% in all treatment combinations, had been stored for two years and appeared to have low quality based on frequent mottled and irregular staining with tetrazolium chloride and exceptionally high mold growth in some germination treatments [13].

If seed availability is high enough, it is often preferable and more cost effective to use direct seeding rather than transplanting in restoration efforts [41]. Seed priming of *I. spuria*, achieved by soaking seeds for seven days in KNO_3_, slightly increased germination [24]. Seed priming of *I. tenax* with warm stratification may prove to be a valuable pre-treatment for fall sowing, allowing the wet cool winter to complete the dormancy breaking process and initiate germination the following spring. We recommend experimental trials of this technique before application in restoration projects.

To create sustainable, stable communities, resistant to invasion, habitat restoration needs to target high diversity[42] including both dominant and uncommon species, because uncommon species can have significant impact on ecosystem function and resiliency [43], [44]. *Iris tenax* is a long-lived, early flowering perennial grassland species and is likely to be included in habitat restoration of prairies throughout Western Oregon and Washington given its importance for rare butterflies and pollinators. Use of this warm-followed-by-cold stratification protocol can substantially improve the efficiency of seed germination and increase the availability of container-grown *I. tenax* plants for use in restoration projects.
